# Spinal Cord Sensitization and Spinal Inflammation from an In Vivo Rat Endplate Injury Associated with Painful Intervertebral Disc Degeneration

**DOI:** 10.3390/ijms24043425

**Published:** 2023-02-08

**Authors:** Alon Lai, Denise Iliff, Kashaf Zaheer, Dalin Wang, Jennifer Gansau, Damien M. Laudier, Venetia Zachariou, James C. Iatridis

**Affiliations:** 1 Leni and Peter W. May Department of Orthopaedics, Icahn School of Medicine at Mount Sinai, New York, NY 10029, USA; 2 Department of Orthopaedics, Nanjing First Hospital, Nanjing Medical University, Nanjing 211166, China; 3 Department of Orthopedic Surgery, University of Kansas Medical Center, 3901 Rainbow Blvd, Kansas City, KS 66160, USA; 4 Nash Family Department of Neuroscience and Friedman Brain Institute, Icahn School of Medicine at Mount Sinai, New York, NY 10029, USA

**Keywords:** endplate injury, intervertebral disc degeneration, spine, spinal cord, central sensitization, macrophage, in vivo, animal model, inflammation, neuroinflammation

## Abstract

Intervertebral disc (IVD) degeneration with Modic-like changes is strongly associated with pain. Lack of effective disease-modifying treatments for IVDs with endplate (EP) defects means there is a need for an animal model to improve understanding of how EP-driven IVD degeneration can lead to spinal cord sensitization. This rat in vivo study determined whether EP injury results in spinal dorsal horn sensitization (substance P, SubP), microglia (Iba1) and astrocytes (GFAP), and evaluated their relationship with pain-related behaviors, IVD degeneration, and spinal macrophages (CD68). Fifteen male Sprague Dawley rats were assigned into sham or EP injury groups. At chronic time points, 8 weeks after injury, lumbar spines and spinal cords were isolated for immunohistochemical analyses of SubP, Iba1, GFAP, and CD68. EP injury most significantly increased SubP, demonstrating spinal cord sensitization. Spinal cord SubP-, Iba1- and GFAP-immunoreactivity were positively correlated with pain-related behaviors, indicating spinal cord sensitization and neuroinflammation play roles in pain responses. EP injury increased CD68 macrophages in the EP and vertebrae, and spinal cord SubP-, Iba1- and GFAP-ir were positively correlated with IVD degeneration and CD68-ir EP and vertebrae. We conclude that EP injuries result in broad spinal inflammation with crosstalk between spinal cord, vertebrae and IVD, suggesting that therapies must address neural pathologies, IVD degeneration, and chronic spinal inflammation.

## 1. Introduction

Chronic back pain is a major global health challenge, affecting 70–85% of the population at some point during life and costing an estimated $134.5 billion in annual US health care expenses [1,2,3]. Intervertebral disc (IVD) pathologies contribute to ~45% of back pain, although back pain can also involve pathological changes to facet joints, sacroiliac joints, spinal muscles and ligaments [4,5]. The sources of painful IVD degeneration are complex and multifactorial, and are known to be highly associated with inflammatory, structural, biochemical and mechanical alterations. Structural defects and pain distinguish pathological IVD degeneration from aging, and structural defects in the endplate (EP) and annulus fibrosus (AF) can cause pain and disability from direct irritation to surrounding neural structures, as well as chronic inflammatory conditions that enable neurovascular ingrowth, sensitization, and heightened nociception. The relatively poor outcomes from spinal surgery for chronic IVD degeneration and the lack of disease-modifying therapies for IVD degeneration prompt a need to understand how spinal defects affect the surrounding nervous system tissues. Relatively few studies investigate the relationship between spinal injuries and spinal cord sensitization, and such knowledge is required to develop improved therapeutic strategies.

Spinal cord injury can result in enhanced nociception via sensitization and neuroinflammation. Spinal cord sensitization can result in increased levels of neuropeptides, such as Substance P (SubP) or CGRP. Spinal cord astrocytes and microglia were also found to be activated and elevated in conjunction with increased pain behavioral responses after peripheral inflammation and nerve injury [6,7,8,9], suggesting neuroinflammation plays a significant role in both inflammatory and neuropathic pain. Microglia are the resident immune cells in the central nervous system, helping to promote clearance of debris, damaged cells, or infectious agents; they are also responsible for proliferation, differentiation, and synaptic hemichannel growth in neurons [10,11]. Astrocytes play important roles, regulating the extracellular environment, supplying nutrients to adjacent nervous tissue, removing excessive neurotransmitters, and modulating endothelial cells that are responsible for maintaining the blood–brain barrier [10]. Importantly, microglia and astrocytes are also responsible for the production of neuroinflammatory mediators, including tumor necrosis factor alpha (TNFα), interleukin-1 beta (IL-1β), chemokine (CC motif) ligand 2 (CCL2), chemokine (CXC motif) ligand 1 (CXCL1) and brain-derived neurotrophic factor (BDNF) [11,12]. Continuous nociceptive input from these mediators following activation of microglia and astrocytes can decrease the threshold of pain and sensitize the spinal cord [13]. While SubP, microglia and astrocytes are known to be important in neuropathic pain, relatively little is known on their roles in chronic discogenic pain.

EP defects are an important contributor to back pain pathologies, as evidenced by the strong clinical relationship between Modic changes and disabling back pain and disability [14,15,16,17]. The lack of effective disease-modifying drugs for EP-driven IVD degeneration points to a need for relevant animal models to improve understanding of the relationship between EP defects, pain and disability. A rat EP microfracture injury ex vivo showed that EP defects result in axial biomechanical instability, while AF defects result in torsional biomechanical instability [18]. We recently established an in vivo EP injury model in rats by puncturing through the vertebral body and EP to the NP of IVD [19]. The EP injuries induced IVD degeneration with decreased IVD height and MRI T2 values, MC type1-like changes on MRI, and enhanced sensory hypersensitivity behaviors. However, the inflammatory changes in the spine and central sensitization in response to EP injury need further elucidation to better understand the pathophysiology of chronic discogenic back pain.

This study investigated if EP injury results in spinal cord sensitization and neuroinflammation, and determined if these pathological spinal cord changes were associated with pain-related behaviors, IVD degeneration, and spine macrophage presence. A surgically induced EP injury was applied in a rat in vivo model developed previously [19]. The objectives of this study were to (1) determine if EP injury triggers the spinal cord with increased substance P (SubP) and results in neuroinflammation with increased microglia (Iba1) and astrocyte (GFAP) markers; (2) determine if EP injury results in macrophage (CD68) accumulation in IVD sub-structures; (3) determine if the resulting spinal cord sensitization and neuroinflammation from EP injury was correlated with the pain-related behavioral responses; and (4) determine if the spinal cord sensitization was correlated with IVD degeneration, and spinal macrophages. Rats were used as a model for EP injury due to their sufficiently large size which allows for precise surgical interventions to induce injury, anatomical similarities to the human spine which make them widely used in studying pathophysiology of IVD degeneration and discogenic pain [20], and because of the availability of assays to assess and quantify their pain-like behavioral responses [21,22,23,24]. EP injury groups included injections with both PBS or TNFɑ, since our rat in vivo study with AF puncture injury showed that TNFɑ injection caused greater severity of pain-related behaviors (sensory hypersensitivity) [25]. Analyses were performed at 8 weeks after EP injury to ensure stable and chronic pain-related behaviors.

The significance of this study is to improve understanding of how EP injury can result in spinal inflammation and spinal cord sensitization in order to help inform future therapeutic strategies.

## 2. Results

### 2.1. Sham and EP Injury Procedures Did Not Affect Rat General Health

**Figure 1:**
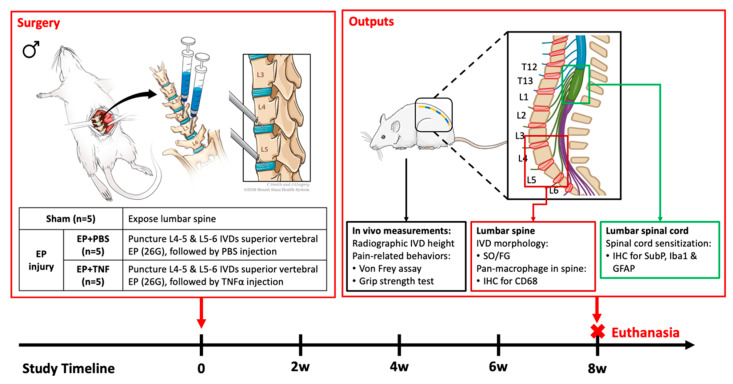
Schematic image of EP injury with anterior approach surgery. Surgical methods and study design [19] with sham (*n* = 5); EP + PBS (*n* = 5) and EP + TNF (*n* = 5), output measurements and timeline.

Fifteen skeletally mature male Sprague Dawley rats (5–6 months old) were used and randomly assigned into one of the three experimental groups (*n* = 5/group): sham (i.e., control), EP + PBS and EP + TNF. EP injury was induced in both EP + PBS and EP + TNF groups by puncturing the EP of L4-5 and L5-6 IVDs from the adjacent vertebral body, followed by intradiscal injection of PBS or TNFα, respectively (Figure 1). There were 2 EP injury groups with L4-5 and L5-6 IVDs punctured obliquely through with a transcorporeal injury through the L5 and L6 vertebral bodies, and through the EP, followed by intradiscal injection of PBS or TNFα (Section 4.1 and Section 4.2). The results from the EP injuries groups were compared with the sham control group which underwent sham surgery to expose the lumbar spine only, without any spine injury. The rat mean ± SD body weights were 561 ± 34 g before surgery, and 568 ± 33 g and 611 ± 35 g at 2 and 8 weeks after surgery, respectively. There were no significant differences in body weight between groups at each time point (*p* > 0.05), indicating the procedures of sham surgery and EP injury were well tolerated by the rats. Body weight slightly increased after surgery for all groups. Prior to surgery, the spine levels were preliminarily identified using a pre-operative anteroposterior radiograph, and the locations of puncture injuries were confirmed using an intra-operative C-arm fluoroscopy. The images of C-arm fluoroscopy indicated that all the needles were properly punctured into the L4-5 and L5-6 IVDs obliquely through the L5 and L6 vertebral bodies, respectively. There were also no intraoperative complications or obvious stress/discomfort from the general physical examination.

### 2.2. EP Injuries Induced Sensory Hypersensitivity with Decreased Hindpaw withdrawal threshold and Peak Grip Force

**Figure 2:**
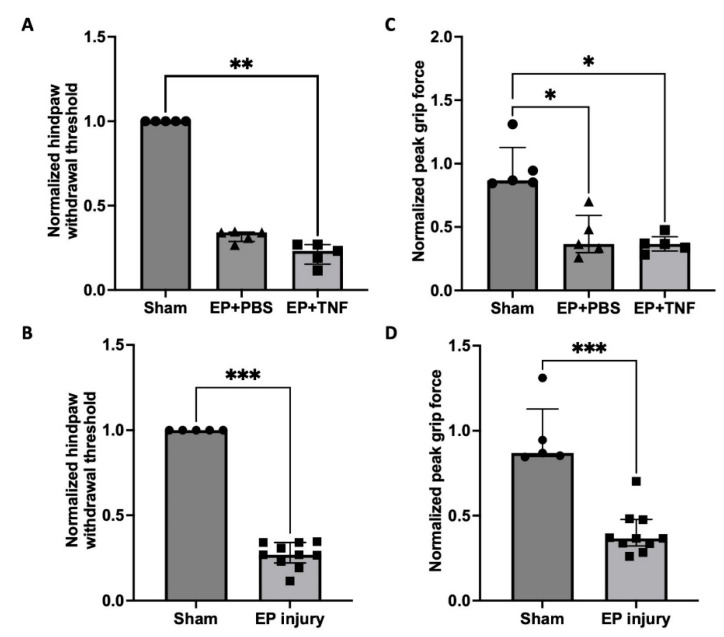
EP injury increased sensory hypersensitivity behaviors, demonstrating mechanical allodynia and axial spinal discomfort with no effect of injectate. (**A**) EP injury with TNFα injection decreased the normalized hindpaw withdrawal threshold. (**B**) Combined EP injury decreased the normalized hindpaw withdrawal threshold. (**C**) EP injury with PBS and TNFα injections decreased the normalized peak grip force. (**D**) Combined EP injury decreased the normalized peak grip force. * *p* < 0.05. ** *p* < 0.01. *** *p* < 0.001.

The normalized hindpaw withdrawal threshold and peak grip force of EP + TNF group were significantly lower than those of the sham group (*p* < 0.05; Figure 2A,C). Both the normalized hindpaw withdrawal threshold and the peak grip force of EP + PBS group were also lower than those of the sham group, but the difference was statistically significant for peak grip force (*p* < 0.05; Figure 2C). There was no significant difference between EP + PBS and EP + TNF groups for both the paw withdrawal threshold and peak grip force (*p* = 0.3893 and *p* > 0.9999, respectively). When combining the 2 EP injuries into one EP injury group, the EP injury group showed significantly lower paw withdrawal threshold and peak grip force than those of the sham group (*p* < 0.001; Figure 2B,D). The results suggested that the EP injuries increased the mechanical sensitivity and spinal discomfort of the rats.

### 2.3. EP Injuries Induced IVD Degeneration with Increased IVD Height Loss and Degeneration Score

**Figure 3:**
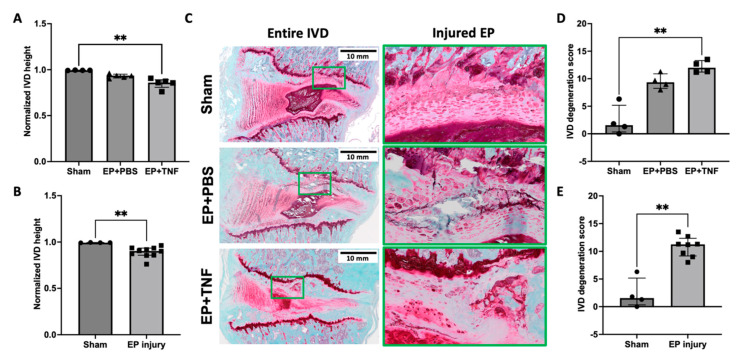
EP injury induced IVD height loss and IVD degeneration. (**A**) EP injury with TNFα injection decreased normalized IVD height. (**B**) Combined EP injury decreased normalized IVD height. (**C**) Representative histological images of entire IVD and highlighted injured EP region (green box) from different experimental groups. (**D**) EP injury with TNFα injection exhibited higher IVD degeneration score. (**E**) Combined EP injury showed higher IVD degeneration score. ** *p* < 0.01.

The normalized IVD height of the EP + PBS and EP + TNF groups was smaller than the sham group, but the difference was statistically significant for the EP + TNF group only (*p* < 0.01; Figure 3A). There was no significant difference between EP + PBS and EP + TNF groups (*p* = 0.2463), and the IVD height of the combined EP injury group was significantly smaller than that of the sham group (*p* < 0.01; Figure 3B).

The morphology of injured L4-5 and L5-6 IVDs following EP injuries was compared with that of the sham group to determine the effects of EP injuries on IVD degeneration. The IVDs following sham surgery showed normal IVD morphology, while EP injuries with either PBS or TNFα injection induced moderate to severe IVD degenerative changes, including shrinkage of NP, less distinct NP-AF boundaries, observable EP disruptions at the injured side consisting more of and clustered EP cells with altered morphology, and herniated NP into the adjacent vertebra through the injured track (Figure 3C). The severity of IVD degeneration was quantified using a degeneration score specifically for rat IVDs. The IVD degeneration score evaluated the NP morphology, NP cellularity, NP-AF border, AF morphology, and EP irregularity, using a score between 0 and 2 for each sub-category, with 0 for normal morphology and 2 for severe degenerative characteristics (Section 4.5, [26]). Our quantitative analysis showed that the IVD degeneration scores were higher in both EP + PBS and EP + TNF groups compared to the sham group, but the difference was statistically significant for the EP + TNF group only (*p* < 0.01; Figure 3D). There was no significant difference between EP + PBS and EP + TNF groups (*p* = 0.6048). The degeneration score of the combined EP injury group was significantly higher than that of the sham group (*p* < 0.01; Figure 3E).

### 2.4. EP Injuries Induced Spinal Cord Sensitization and Neuroinflammation

**Figure 4:**
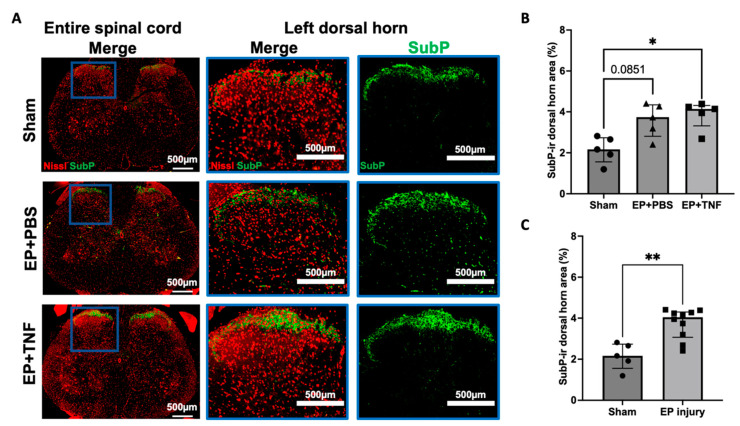
EP injury increased SubP in spinal dorsal horn with no effect of injectate. (**A**) Representative images of SubP immunohistochemical staining with Nissl stain in the entire spinal cord and left dorsal horn. (**B**) EP injury with PBS and TNFα injections increased SubP-ir in the spinal cord dorsal horn. (**C**) Combined EP injury increased SubP-ir in the spinal cord dorsal horn. * *p* < 0.05. ** *p* < 0.01.

SubP is an important nociceptive neurotransmitter in the nervous system. The SubP immunoreactivity (ir) was mainly located at the lamellae I and II of the spinal cord (Figure 4A), which are the terminations of nociceptive A-delta and C fibers, suggesting an activation of nociceptors A-delta and C fibers. The percentage of SubP-ir was higher in both EP + PBS (*p* = 0.0851) and EP + TNF (*p* < 0.05) groups when compared to sham (Figure 4B). Since there was no significant difference between the 2 EP injury groups (*p* > 0.9999), the EP + PBS and EP + TNF groups were combined as an EP injury group and compared with sham to determine the effects of EP injuries on spinal cord sensitization. The SubP-ir of the EP injury group was significantly higher than that of sham group (*p* < 0.01; Figure 4C). The increase in SubP reveals central sensitization with neuronal hyperactivity and hyperexcitability in spinal cord, which might be associated with the increased behavioral hypersensitivity.

**Figure 5:**
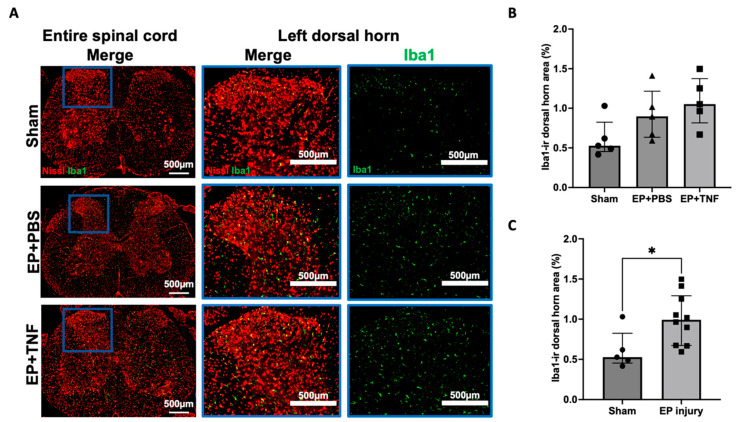
EP injury increased Iba1 in the spinal dorsal horn with no effect of injectate. (**A**) Representative images of Iba1 immunohistochemical staining with Nissl stain in the entire spinal cord and left dorsal horn. (**B**) EP injury with PBS and TNFα injections slightly increased SubP-ir in the spinal cord dorsal horn, but this was not statistically significant. (**C**) Combined EP injury increased Iba1-ir in the spinal cord dorsal horn. * *p* < 0.05.

**Figure 6:**
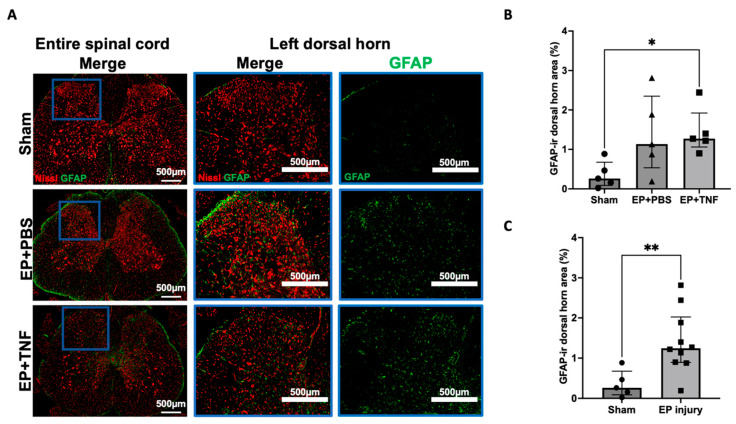
EP injury increased GFAP in the spinal dorsal horn with no effect of injectate. (**A**) Representative images of GFAP immunohistochemical staining with Nissl stain in the entire spinal cord and left dorsal horn. (**B**) EP injury with TNFα injection increased GFAP-ir in the spinal cord dorsal horn. (**C**) Combined EP injury increased GFAP-ir in the spinal cord dorsal horn. * *p* < 0.05. ** *p* < 0.01.

Iba1 and GFAP are markers for activated microglia and astrocytes, respectively, which are important glial cells in the spinal cord. Iba1 and GFAP-ir were evenly distributed in the spinal cord dorsal horn (Figure 5A and Figure 6A). Compared to the sham group, the percentages of Iba1-ir and GFAP-ir were relatively higher in both EP + PBS and EP + TNF groups, but only GFAP-ir showed a significant difference between EP + TNF (*p* < 0.05; Figure 6B). Similar to SubP-ir, there was no significant difference in Iba1- and GFAP-ir between the EP + PBS and EP + TNF groups (both *p* > 0.9999). The EP + PBS and EP + TNF groups were combined as an EP injury group and compared with sham, and both the Iba1- and GFAP-ir of the combined EP injury group were significantly higher than those of the sham group (*p* < 0.05 and *p* < 0.01, respectively; Figure 5C and Figure 6C). The activated spinal microglia and astrocytes promote the secretion of glial mediators, including cytokines and chemokines, which can act as neuromodulators to induce central sensitization by modulating both excitatory synapses and inhibitory synapses.

### 2.5. EP Injury Increased Macrophage Levels in EP and Vertebral Body

**Figure 7:**
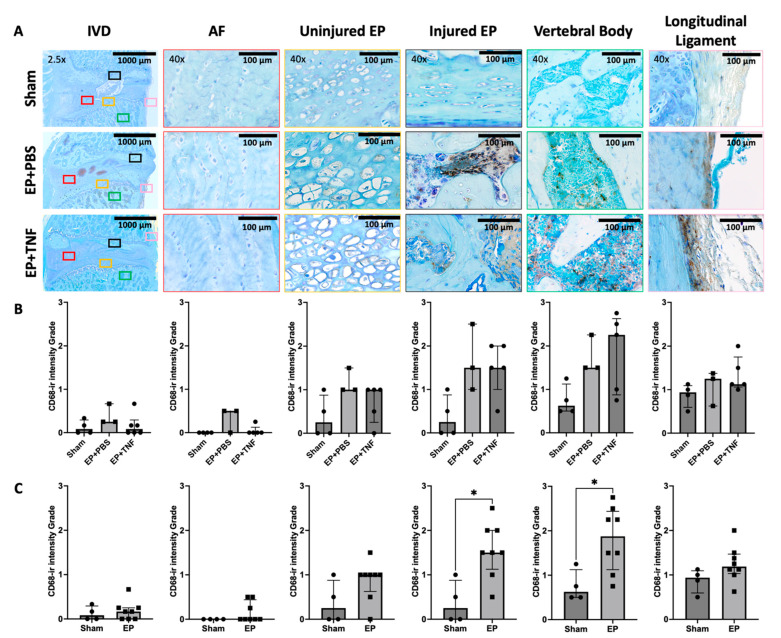
EP injury increased CD68 activity in the spine. (**A**) Representative images of CD68 immunohistochemical staining with toluidine blue in entire IVD, AF, injured and uninjured EP, vertebral body, and longitudinal ligament. (**B**) Semi-quantitative analysis showed EP with PBS and TNFα injections slightly increased CD68-ir in the injured EP and vertebral body, but not to a statistically significant extent. (**C**) Combined EP injury increased CD68-ir in the injured EP and vertebral body. * *p* < 0.05.

**Figure 8:**
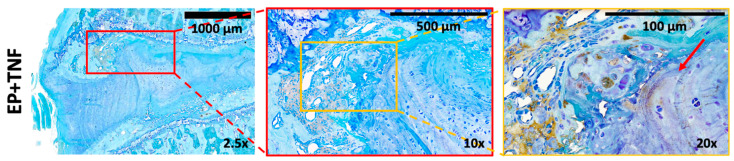
EP injury increased CD68-ir in vertebral and cartilage EP cells at the injury site. Representative EP+ TNFα images of CD68 immunohistochemical staining with toluidine blue showing disrupted EP structure with intense CD68-ir in the vertebral and cartilage EP regions in the injured EP in proximity to the injury site. There is also a lack of structural demarcation between cartilage and vertebral EP in proximity to the injury site. Red arrow indicates the injury site.

CD68 is a marker for inflammation associated with the involvement of monocytes or macrophages. There was some CD68-ir in the vertebral body and longitudinal ligaments of the sham rat spines. There was relatively more CD68-ir in the spines of the rats following EP injuries, mainly in the regions of the injured EP and vertebral body; particularly intense staining was located at EP and vertebral bone adjacent to the injured region (Figure 7A and Figure 8). Some CD68-ir was also observed in the uninjured side of EP and longitudinal ligaments; however, there was very little CD68-ir in the IVD or AF (Figure 7A). In the injured EP, CD68-ir was particularly strong in the injured EP in proximity to the injury site, whereas CD68-ir was observed in vertebral and cartilage EP regions, and there was no longer clear structural demarcation between cartilage and vertebral EP regions (Figure 8).

CD68-ir was quantified using a semi-quantitative scoring system ranging from 0 to 3 for the whole IVD, and for each IVD and vertebral sub-region (Supplementary Figure S1). The CD68-ir was higher in the injured EP and vertebral body in both the EP + PBS and EP + TNF group compared to the sham group, but the differences were not statistically significant (*p* > 0.05; Figure 7B). There was no statistical significance between the EP + PBS and EP + TNF groups (both *p* > 0.9999); therefore, they were combined and compared with the sham group. We found that the CD68-ir in the regions of the injured EP and vertebral body of the combined EP injury group was significantly higher than that of the sham group (both *p* < 0.05; Figure 7C). There was no statistically significant difference in CD68-ir between the EP injury and sham groups for other regions of the spine.

### 2.6. Spinal Cord SubP-Ir Correlates with GFAP-Ir

Spearman correlation analysis showed that the percentage of SubP-ir was significantly and positively correlated with the percentage of GFAP-ir (R = 0.661, *p* = 0.009) (Table 1), suggesting there was an association in the level and/or production between SubP and GFAP at this chronic time point. However, the percentage of Iba1-ir was not correlated with either SubP-ir nor GFAP-ir, suggesting the level of microglia (Iba1) was independent of SubP and astrocytes (GFAP).

### 2.7. Spinal Cord SubP-, Iba1- and GFAP-Ir, IVD Degeneration as Well as EP and Vertebral CD68-Ir Correlate with Pain-Related Behaviors

Correlation analyses also showed that the pain-related behaviors of grip peak force and paw withdrawal threshold were significantly correlated with spinal cord SubP-, Iba1- and GFAP-ir, IVD height, IVD degeneration score and CD68-ir at injured EP (all *p* < 0.05; Table 2), suggesting spinal cord SubP, Iba1 and GFAP as well as IVD degeneration and inflammation at injured EPs might be important for the presence of pain-related behaviors at 8 weeks after EP injury. Moreover, the grip peak force was also correlated with CD68-ir at the uninjured EP (*p* < 0.05), vertebral body and longitudinal ligaments (both *p* < 0.1), suggesting that spine discomfort might be (partially) associated with spine inflammation.

### 2.8. Spinal Cord Sensitization and Neuroinflammation Correlate with IVD Degeneration and Spine Inflammation

The percentages of SubP-, Iba1- and GFAP-ir were significantly correlated with IVD height and IVD score (all *p* < 0.05, except *p* < 0.1 for Iba1-ir with IVD height; Table 3). The results suggest there is crosstalk between the IVD and spinal cord, and that the IVD degeneration resulting from EP injury might induce spinal cord sensitization and neuroinflammation. Moreover, SubP- and Iba1-ir were correlated with CD68-ir in the injured EP (*p* < 0.05 for SubP and *p* < 0.1 for Iba1) and vertebral body (*p* < 0.05 for Iba1 and *p* < 0.1 for SubP), suggesting there was association between spinal inflammation and spinal cord sensitization.

## 3. Discussion

This is the first study to demonstrate that EP injury in rats induces spinal cord sensitization and neuroinflammation. EP injury was previously shown to induce Modic-like changes and IVD degeneration and result in increased pain-like behaviors [19]. This study demonstrated EP injury increased SubP, Iba1 (microglia) and GFAP (astrocytes) in the dorsal horn of the spinal cord at 8 weeks after injury. EP injury significantly increased multiple inflammatory cell markers including Iba1 and GFAP in the spinal cord, and CD68 (macrophage) in the EP and vertebral body regions, demonstrating inflammatory activity in multiple spinal tissues. EP injury increased pain-related behaviors, with a decreased hindpaw mechanical withdrawal threshold and axial grip force; additionally, the changes of behavioral responses were significantly correlated with spinal cord SubP-, Iba1- and GFAP-ir as well as IVD height, IVD degeneration and CD68-ir in injured EP. Overall, these results indicate that EP injury can cause a complex pathology involving SC sensitization, neuroinflammation, and IVD degeneration, and all of these factors can contribute to increased nociceptive responses. EP injury increased inflammatory cells in the spinal cord, EP and vertebral body, demonstrating crosstalk between all of these spinal tissues. We therefore conclude that EP injury can cause broad spinal inflammation, and treatment strategies for chronic discogenic pain may likely require interventions targeting both spinal and neural pathologies.

EP injury increased the pain-related behaviors that were significantly correlated with spinal cord SubP-, Iba1-, and GFAP-ir, indicating the important role that spinal cord sensitization plays in chronic discogenic pain. SubP is a neurotransmitter for transmitting nociceptive signals. In this study, spinal cord SubP was mainly localized to laminae I and II (Figure 2), where terminations of nociceptive A-delta and C nerve fibers reside [27]. AF-driven IVD degeneration (i.e., AF puncture injury) showed that SubP was increased in L2 dorsal root ganglia after AF injury [25], suggesting SubP is involved in both the peripheral and central nervous systems’ responses to a variety of spine injuries that cause IVD degeneration. The increased Iba1 and GFAP in the spinal dorsal horn in this study were similar to the changes in the spinal cord following nerve injury which has been previously shown to increase and activate astrocytes and microglia, thereby contributing to increased nociception in neuropathic pain [6,7,8,9]. We infer that EP injury induces spinal cord sensitization and neuroinflammation, which have some similarities to neuropathic conditions. A kinetics study on peripheral nerve injury also showed microglia were increased at earlier time points (i.e., days 7 and 14) post-injury, while astrocytes were increased at chronic time points (i.e., days 22 and 150) post-injury; this is consistent with the hypothesis that microglia are involved with onset of pain hypersensitivity, while astrocytes play roles in the maintenance of chronic pain. These findings together may encourage the performance of future studies with early and chronic time points to further test this hypothesis with EP and other IVD injuries, and to identify if early and late responses require distinct treatment strategies.

EP injury appears to result in broad inflammatory crosstalk between the spinal cord, vertebrae and IVD. EP injury increased the inflammatory cell markers in spinal cord, EP, and vertebrae, and spinal cord markers (SubP-, Iba1- and GFAP-ir) which were correlated with EP and vertebral body CD68-ir levels, revealing an inflammatory crosstalk between the IVD, vertebrae and spinal cord. Our study showed direct crosstalk between the vertebrae and IVD in close proximity to the injury site wherein there was structural disruption and CD68-ir in vertebral and cartilage EP cells, suggesting macrophage infiltration in vertebral and IVD structures (Figure 7). While we did not find that CD68-ir increased from EP injury, the spinal cord markers correlated to IVD height and IVD degeneration, suggesting other pro-inflammatory markers and earlier time points, are likely to be important in characterizing IVD inflammation following EP injury. Furthermore, spinal cord and IVD crosstalk is also suggested by AF puncture injury and aged SPARC (secreted protein, acidic, rich in cysteine)-null mice which showed increased astrocytes in the spinal cord, IVD degeneration, and pain-related behaviors [28,29]. Miyagi et al. further showed AF injury significantly increased intradiscal TNFα at 1, 4 and 7 days post injury, and significantly increased microglia and astrocytes in the spinal cord at 1 week and 8 weeks post injury [28]. Overall, taking into account the literature, we believe EP injury causes inflammatory crosstalk between the spinal cord, IVD and vertebrae that might initiate spinal cord sensitization. The broad spine inflammatory responses from EP injury, therefore, might suggest that anti-inflammatory treatments may need to target spinal cord, IVD and vertebrae to prevent or reduce EP-driven discogenic pain.

Significant correlations between SubP-ir and GFAP-ir in the spinal cord indicated that nociceptive neurotransmitters may be associated with spinal cord astrocytes. SubP was shown to play a mechanistic role in spinal cord microglia and astrocyte activation as well as nociception in a rat tibial fracture model [30]. Microglial-derived TNFα can also activate nearby astrocytes [11]. However, the literature of spinal cord markers in discogenic pain models is sparse, and more research with a wider spectrum of spinal cord pain-related markers at acute and chronic time points in discogenic pain is needed to better understand the specific roles of these markers and to identify potential therapeutic strategies. Nevertheless, these results and the literature suggest spinal cord SubP, astrocytes and microglia play complex and interacting roles in nociception, and suggest that SubP inhibitors are potential targets for discogenic pain and neuroinflammation.

EP injury was more important than the injectate type (PBS vs. TNFα injection) in driving the observed molecular changes in the spinal cord or pain-related behaviors in this study. Specifically, no significant differences between PBS or TNFα injection were detected in spinal cord markers or pain-related behaviors, although some effects of a ‘dose–response’ were suggested for IVD height and IVD degeneration grade. Interestingly, AF-puncture injury in rats had similar effects on IVD degeneration and SubP in the L2 dorsal root ganglia with either PBS or TNFα injection, although TNFα injection induced more sensory hypersensitivity than PBS injection [25]. Together, these studies suggest that maintaining the integrity of the spinal structure is critical. We believe the dominant effect of EP injury over injection type is because a single TNFα injection may have little effect beyond the baseline pro-inflammatory cytokines present from the injury, since adult IVDs heal poorly and IVD degeneration is known to result in chronic pro-inflammatory conditions [31,32,33].

Some limitations of this study are important to highlight. The sample size was sufficient to identify statistically significant differences between sham and EP injury groups for all variables tested, to identify many significant correlations, and to achieve our objectives to induce severe degenerative changes that mimic the human condition. While results did not suggest greater severity of changes for EP+ TNFα vs. EP + PBS groups, a higher sample size would provide more definitive findings to detect potential differences between these groups, and we therefore present statistical analyses with both 2-group and 3-group comparisons to allow the reader to assess these differences. We also cannot determine the effects of EP injury without any injection, so we did not use a control group of EP injury without any injection. We also note that the PBS injection volume was small, and slowly injected into the IVD (~10 s for 2.5 µL) during surgery to allow the injected PBS to be absorbed by the IVD without inducing visible IVD protrusion. We therefore expect that the EP + PBS group in this study is likely to be roughly comparable to EP injury without any injections. An increased sample size might also enable more definitive conclusions regarding changes in Iba1 with EP injury. However, previous studies report that spinal cord microglia play more prominent roles in pain hypersensitivity at early time points following spinal cord injury [7], which is consistent with the limited changes observed in Iba1 cells at the chronic time point of 8 weeks after EP injury. We prioritized the chronic 8 week time point in this study which we believe is most relevant to the human patient cohort and which is most commonly a chronic condition. We note, however, that this rat EP injury is an acute injury, unlike human IVD degeneration with EP changes that accumulate over years or decades and are more prominent in aged populations. Male rats in this study showed spinal cord sensitization and strong behavioral changes following EP injury. Pain mechanisms, neuroinflammation, and IVD healing responses can differ between males and females [24,34,35,36]. Future studies in female rats will be important to identify whether spinal cord sensitization following EP injury differs between males and females.

## 4. Materials and Methods

### 4.1. Study Design

All experimental procedures were guided and approved by the Institutional Animal Care and Use Committee. Fifteen skeletally mature male Sprague Dawley rats (5–6 months old) were used and randomly assigned into one of the three experimental groups (*n* = 5/group): sham, EP + PBS and EP + TNF. EP injury was induced in both EP + PBS and EP + TNF groups by puncturing the EP of L4-5 and L5-6 IVDs from the adjacent vertebral body, followed by intradiscal injection of PBS or TNFα, respectively (Figure 1). The sham group is the control group for determining the effect of surgery without EP injuries, while EP injury groups included PBS or TNFα injection (Section 4.2). Surgery was performed to expose the lumbar spine of vertebral bodies between L4-L6, as well as L4-5 and L5-6 IVDs, without any puncture or injection. The IVD height and pain-related behaviors were measured before and at 8 weeks after injury in vivo. All the animals were euthanized at 8 weeks post-surgery with transcardial perfusion of 10% buffered formalin phosphate (Fisher Company, Fair Lawn, NJ, USA) under the condition of anesthetization. The L4-6 lumbar spine and lumbar spinal cord (corresponding to T12-L1 vertebral level) were dissected and further fixed in 10% buffered formalin phosphate for at least 48 h. The formalin-fixed spine was used for post-mortem MRI and microCT followed by histological analysis, while the lumbar spinal cord was used for immunohistochemical analysis for SubP, Iba1 (microglia) and GFAP (astrocyte).

### 4.2. Surgical Procedure for EP Injury

Surgical procedures were performed under aseptic conditions, with the rat under general anesthesia, using 2% isoflurane in oxygen (Baxter, Deerfield, IL, USA). The rat lumbar spine was accessed using an anterior approach with a midline incision at the abdominal wall. The spine levels were preliminarily identified using a pre-operative anteroposterior radiograph and confirmed using an intra-operative C-arm fluoroscopy. The proximal EPs of L4-5 and L5-6 IVDs were punctured obliquely from the L5 and L6 vertebral bodies at 1.5 mm proximal to the edge of the IVDs, using a 0.6 mm K-wire at a depth of 3 mm, which was guided by a stopper (Figure 1). A calibrated microliter syringe (Hamilton Company, Reno, NV, USA). A 26-gauge needle was then placed along the injury track at a depth of 3 mm, guided by a needle stopper [19]. A total of 2.5 μL of PBS or TNFα (0.25 ng in 2.5 μL) (80045RNAE50; Sino Biological Inc., Beijing, China) was then slowly injected into each IVD [22,24,25,36]. All EP injuries were guided and confirmed radiologically using the C-arm fluoroscopy. The peritoneum was closed using 3–0 silk sutures, and the skin was closed using 4–0 nylon sutures.

Following a 24 h post-surgery single-rat isolation period, the rats were allowed unrestricted movement in their home cages (2 rats per cage) for the remaining duration of the 8 week experiment. They were also allowed ad libitum access to food and water, and closely monitored to ensure no intraoperative complications.

### 4.3. Pain-Related Behavior Measurements

Pain-related behaviors were evaluated using the von Frey assay for mechanical allodynia at the hindpaws [19,22,23,24,25,36,37] and a grip strength test for axial spine discomfort [19,29,37,38]. All the behavioral measurements were performed by a single experimenter in a dedicated behavioral analysis room with regular indoor lighting.

For the von Frey assay, test cages with a wire-mesh floor were used to allow access to the hindpaw from below. Prior to testing, all the rats were acclimated to handling and resided in their test cages for 7 consecutive days. On the day of testing, after 20 min of acclimation, the calibrated von Frey filaments were applied to the plantar surface of each hindpaw in ascending order from 0.4 to 26.0 g repeating five times for each filament. The lowest filament eliciting nocifensive behaviors, including paw licking, extended paw withdrawal, and fanning/shaking of the paw in 3 out of 5 applications was considered the paw withdrawal threshold. The paw withdrawal thresholds from the left and right hindpaws were averaged for statistical analysis. A decreased paw withdrawal threshold was considered increased pain sensitivity.

For the grip strength test, a custom-built testing apparatus with a stainless steel grid connecting to a uniaxial force sensor was used. The rat was positioned with forepaws grabbing the metal grid. The spine of the animal was then stretched by gently pulling its tail until it released from the grid. The peak grip force was measured and recorded by a force sensor using LabVIEW software (National Instruments Corporation, Austin, TX, USA). A total of three trials were performed, with a 10 min break between trials. The results from the three trials were averaged for statistical analysis. A decreased grip force was considered representative of increased axial spine discomfort.

### 4.4. Radiographic IVD Height

The heights of the lumbar IVDs were assessed using a lateral radiograph in vivo, with the animals under general anesthesia, using inhaled isoflurane [22,23,24,25,36,37]. The animal was radiographed using standardized protocol (UltraFocus Faxitron, Tucson, AZ, USA) after a 10 min anesthetization period in order to minimize the effect of anesthesia on IVD height resulting from muscle relaxation and IVD swelling. The vertebral boundaries of L4-5 and L5-6 IVDs were manually identified, and the IVD height was calculated using a custom MATLAB script (MathWorks, Natick, MA, USA). The height of the L4-5 and L5-6 IVDs was averaged, and the results from 8 weeks post-surgery were normalized to those from pre-surgery to obtain the percentage change.

### 4.5. Immunohistochemical Analysis for Spinal Cord Sensitization

The formalin-fixed spinal cord was embedded in paraffin and sectioned sagittally at 5μm intervals. Two sections per animal, spread across the lumbar spinal cord, were selected for each antibody. After deparaffinization and rehydration, the spinal cord sections were treated an with antigen-retrieval buffer of Histo/zyme (H3292, Sigma-Aldrich, Inc, St. Louis, MO, USA) followed by a protein-blocking buffer of 2.5% normal horse serum (Vector Laboratories, Inc, Burlingame, CA, USA). The sections were then incubated at room temperature for 1 h with mouse monoclonal primary antibody against rat SubP (1:300 dilution, ab14184, Abcam, Cambridge, MA, USA), rabbit monoclonal Iba1 (1:1000 dilution, ab178846, Abcam, Cambridge, MA, USA), rabbit polyclonal primary antibody against rat GFAP (1:2000, ab7260, Abcam, Cambridge, MA, USA), or negative control with normal mouse serum (NC494H, Biocare Medical, LLC, Pacheco, CA, USA) or normal rabbit serum (NC495H, Biocare Medical, LLC). A secondary antibody for SubP was carried out with a VectaFluor Excel Amplified anti-mouse IgG, Dylight 488 antibody kit (DK-2488, Vector Laboratories, Inc, Burlingame, CA, USA), and for Iba1 and GFAP with a VectaFluor Excel Amplified anti-rabbit IgG, Dylight 488 antibody kit (DK-1488, Vector Laboratories, Inc, Burlingame, CA, USA) according to the instructions from the manufacturer. Briefly, after primary antibody incubation and buffer washing, the slides were incubated with amplifier antibody followed by Dylight488 VectaFluor reagent to visualize the immunoreactivity. The sections were then incubated with NeuroTrace 530/615 red fluorescent Nissl stain (1:200 dilution, N21482, Thermo Fisher Scientific, Waltham, MA, USA) to visualize the neurons and glial cells. All steps were performed at room temperature and in a dark, moist chamber. The stained slides were mounted using a ProLong™ Gold Antifade Mount with DAPI (P36931, Thermo Fisher Scientific, Waltham, MA, USA), and imaged using a Leica DM6 B microscope (Leica Microsystems, Inc, Deerfield, IL, USA) at 20x magnification with standardized microscope settings. All images were analyzed using ImageJ. A standardized immunoreactivity (ir) threshold was applied to all images, and the percentage of SubP-, Iba1- and GFAP-ir relative to area of spinal dorsal horn was quantified and then averaged between the left and right dorsal horn as well as across the two sections from each animal.

### 4.6. IVD Morphology

**Figure 9:**
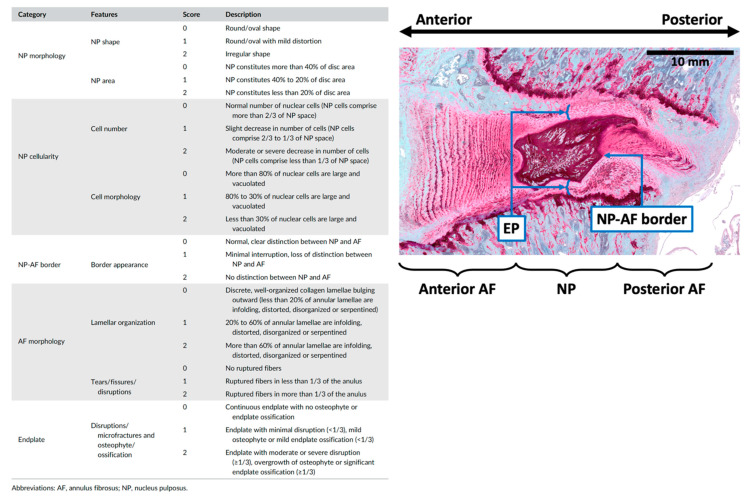
Degeneration grading system with five sub-categories for quantifying the severity of rat IVD degeneration (adopted from Lai et al. JOR Spine 2021 [26]). Rat IVD, with safranin-O/Fast-green/Hematoxylin staining indicating different IVD regions for IVD degeneration quantification.

After MRI scanning, the fixed specimens were decalcified, embedded in resin, and sectioned sagittally at 5 μm intervals. The midsagittal section with the EP injury was then identified. After de-plasticization and re-hydration, the sections were stained with Safranin-O/fastgreen/hematoxylin (SO/FG/H) for disc morphology, glycosaminoglycan (GAG) content, and IVD cellularity. The stained sections were imaged using bright field microscopy (Leica Microsystems, Inc, Deerfield, IL, USA). The severity of IVD degeneration was quantified using a grading system that evaluated NP morphology, NP cellularity, NP-AF border, AF morphology, and EP irregularity [26] (Figure 9). All spine images were evaluated by three evaluators who were blinded to the experimental groups. The degeneration scores from the three evaluators were averaged for statistical analysis.

### 4.7. Immunohistochemical Analysis for Pan-Macrophage in Spine

The expression of pan-macrophage in the spine was identified using immunohistochemistry for CD68. The mid-sagittal sections with EP disruption were identified, followed by de-plasticization and re-hydration. After antigen retrieval with IHC Enzo (ADI950-280-0015, ENZO Life Science, Farmingdale, NY, USA) and protein block (X0909, Agilent Technologies, Inc., Santa Clara, CA), the sections were incubated for 1 h at room temperature with rabbit monoclonal primary antibody against rat CD68 (1:1000 dilution, ab125212, Abcam, Waltham, MA, USA) or normal rabbit serum (S-500-20, Vector Laboratories, Inc, Burlingame, CA, USA) as the negative control. After incubation with horseradish peroxidase-conjugated anti-rabbit secondary antibody (MP-7061, Vector Laboratories, Inc, Burlingame, CA, USA), the sections were treated with diaminobenzidine-based peroxidase substrate (MP-7601, Vector Laboratories, Inc, Burlingame, CA, USA) to visualize the immunoreactivity. The sections were then counterstained with toluidine blue for spine morphology, dehydrated, mounted, and evaluated using a bright field light microscope (Axio Imager. Z1 microscope, Zeiss, Thornwood, NY, USA). A semi-quantitative grading scale was established to semi-quantitatively assess the CD68-immunoreactivity (-ir) in the rat spine. The grading scale was between 0 and 3, with grade 0 for no CD68-ir, 1 for little CD68-ir, 2 for moderate CD68-ir, and 3 for intense staining of CD68-ir. The CD68-ir in NP, AF (anterior and posterior), EP (proximal and distal), vertebral body (proximal and distal), as well as longitudinal ligaments (anterior and posterior) at the levels of L4-5 and L5-6 (i.e., the injured spine) were evaluated by researchers who were blinded to the experimental groups. The CD68-ir from the two spine levels, the anterior and posterior regions of AF and longitudinal ligaments, as well as injured and uninjured regions of EP and vertebral body were averaged for statistical analysis. The gradings from anterior and posterior AF as well as NP were also averaged to determine the overall grading of the entire IVD for statistical analysis.

### 4.8. Statistical Analysis

The IVD heights, hindpaw withdrawal thresholds and peak grip forces obtained at 8-week post-surgery were normalized to pre-surgery and presented as percent change to minimize individual variability. The normalized IVD height, hindpaw withdrawal thresholds and peak grip forces, IVD degeneration score, as well as SubP-, Iba1-, GFAP- and CD68-ir were analyzed using a non-parametric Kruskal–Wallis test with Dunn’s multiple comparison test. A non-parametric Mann–Whitney test was used to determine the difference between the sham and EP injury (i.e., combined EP + PBS and EP + TNF) groups. A non-parametric Spearman’s correlation test was used to identify the correlations between the percentages of SubP-, Iba1- and GFAP-ir and paw withdrawal threshold, grip strength, IVD height, IVD score and CD68-ir. All statistical analyses were conducted using Prism9 (GraphPad Software, Inc., La Jolla, CA, USA), with the level of significance set at α = 0.05.

## 5. Conclusions

This study demonstrated that EP injuries induced chronic spinal cord sensitization with increased SubP, and neuroinflammatory processes with increased GFAP. EP injury also resulted in chronic pain-related behaviors that were all significantly associated with SubP, Iba1 and GFAP, indicating the strong relationship of pain with spinal cord sensitization and neuroinflammation. Spinal cord markers were also significantly correlated with IVD height, the grade of IVD degeneration, and EP macrophage presence, demonstrating crosstalk between the IVD, vertebrae, and spinal cord following EP injury. The crosstalk between the IVD, vertebrae and spinal cord in EP-driven discogenic pain is likely to require therapies that address neural pathologies, IVD degeneration, and spine inflammation.

## Figures and Tables

**Table 1:** Correlation analysis among spinal cord SubP-, Iba1- and GFAP-ir.

		Iba1	GFAP
SubP	Spearman r	0.250	**0.661**
	*p*-value	0.368	0.009 *
Iba1	Spearman r		0.400
	*p*-value		0.141

* *p* < 0.05.

**Table 2:** Correlation analysis to identify important factors contributing to pain-related behaviors.

		**SubP**	**Iba1**	**GFAP**	**IVD Height**	**IVD Degeneration Score**	
Normalized peak grip force	Spearman r	**−0.769**	**−0.520**	**−0.534**	**0.620**	**−0.611**	
	*p*-value	0.001 *	0.049 *	0.042 *	0.020 *	0.038 *	
Normalized paw withdrawal threshold	Spearman r	**−0.554**	**−0.563**	**−0.539**	**0.875**	**−0.793**	
	*p*-value	0.035 *	0.031 *	0.041 *	0.000*	0.003 *	
		**CD68** **(IVD)**	**CD68** **(AF)**	**CD68** **(Uninjured EP)**	**CD68** **(Injured EP)**	**CD68** **(Vertebral Body)**	**CD68** **(Longitudinal Ligament)**
Normalized peak grip force	Spearman r	−0.368	−0.522	**−0.803**	**−0.736**	−0.596	−0.585
	*p*-value	0.291	0.122	0.008 *	0.018 *	0.073	0.081
Normalized paw withdrawal threshold	Spearman r	0.040	−0.046	−0.434	**−0.647**	−0.603	−0.269
	*p*-value	0.922	0.931	0.211	0.048 *	0.070	0.449

* *p* < 0.05.

**Table 3:** Correlation analysis to identify important factors contributing to spinal cord sensitization.

		**IVD Height**	**IVD Degeneration Score**		**CD68 (IVD)**	**CD68 (AF)**
SubP	Spearman r	**−0.684**	**0.634**		0.125	0.150
	*p*-value	0.009 *	0.030 *		0.738	0.678
Iba1	Spearman r	−0.490	**0.588**		0.059	0.015
	*p*-value	0.078	0.047 *		0.883	0.983
GFAP	Spearman r	**−0.697**	**0.816**		0.151	0.112
	*p*-value	0.007 *	0.002 *		0.681	0.761
		**CD68** **(Uninjured EP)**	**CD68** **(Injured EP)**	**CD68** **(Vertebral Body)**	**CD68** **(Longitudinal Ligament)**	
SubP	Spearman r	**0.750**	**0.749**	0.634	0.448	
	*p*-value	0.017 *	0.016 *	0.054	0.199	
Iba1	Spearman r	0.248	0.582	**0.659**	0.522	
	*p*-value	0.486	0.083	0.044 *	0.129	
GFAP	Spearman r	0.165	0.372	0.616	0.436	
	*p*-value	0.648	0.290	0.063	0.213	

* *p* < 0.05.

## Data Availability

The data that support the findings of this study are available from the corresponding author, James C. Iatridis, upon request.

## Supplementary Materials

The following supporting information can be downloaded at: https://www.mdpi.com/article/10.3390/ijms24043425/s1.
