# Supplementary material for: Spinal Cord Sensitization and Spinal Inflammation from an In Vivo Rat Endplate Injury Associated with Painful Intervertebral Disc Degeneration
Source: Int J Mol Sci. 2023 Feb 8;24(4):3425. doi: 10.3390/ijms24043425 (PMC9964286; doi:10.3390/ijms24043425)
Supplement: Supplementary file 1 [file ijms-24-03425-s001.zip › ijms-2142858-supplementary.pdf]

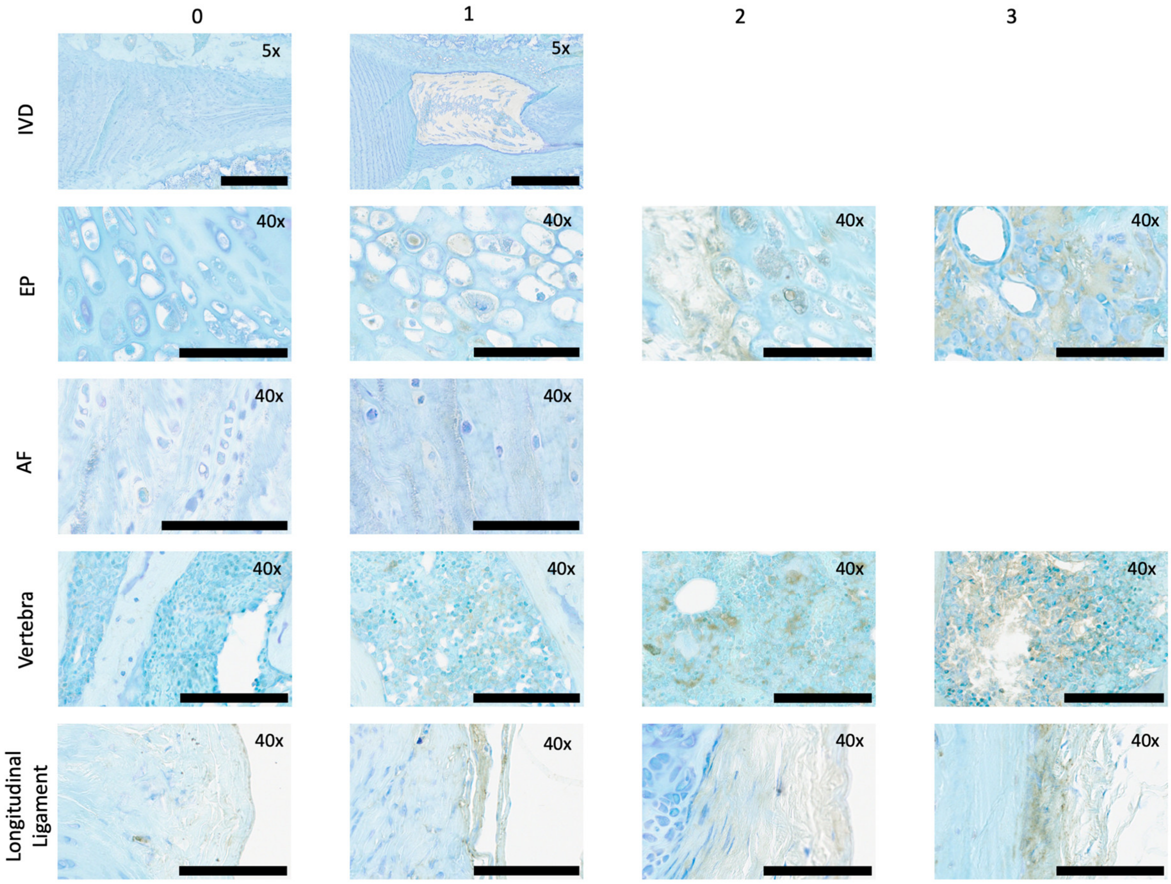

Supplementary Figure S1. Stain intensity scoring system for CD68 immunostaining with representative images. The stain intensity score was applied to regions of interest (ROIs) included the whole IVD, endplate (EP), annulus fibrosus (AF), vertebra, and longitudinal ligament. The semi-quantitative scoring ranged from 0 to 3 and was assigned as: 0 = no staining; 1 = some positive staining; 2 = moderate levels of staining; 3 = extensive staining and/or robust staining in local areas of the ROI. Scale bars = 1000µm and 100µm for 5x and 40x, respectively.
